# Embedding the ‘CoolCuddle’ intervention for infants undergoing therapeutic hypothermia for hypoxic-ischaemic encephalopathy in NICU: an evaluation using normalisation process theory

**DOI:** 10.1136/bmjopen-2024-088228

**Published:** 2024-10-18

**Authors:** Lucy Beasant, Ela Chakkarapani, Jeremy Horwood, David Odd, Stephanie Stocks, Denise Parker, Jenny Ingram

**Affiliations:** 1 Centre for Academic Child Health, University of Bristol, Bristol, UK; 2 University of Bristol, Bristol, UK; 3 Centre for Academic Primary Care, University of Bristol, Bristol, UK; 4 University Hospital of Wales, Cardiff, UK; 5 Neonatal Care Unit, University Hospitals Bristol and Weston NHS Foundation Trust, Bristol, UK

**Keywords:** neonatal intensive & critical care, delivery of health care, integrated, neonatology, patient-centered care, qualitative research, surveys and questionnaires

## Abstract

**Abstract:**

**Objectives:**

Newborn infants exposed to lack of oxygen and blood flow to the brain around birth may develop brain dysfunction (hypoxic-ischaemic encephalopathy—HIE). These infants undergo 72 hours of cooling therapy and most are not held by their parents in the UK. We examined the implementation of ‘CoolCuddle’, identifying factors that impact embedding of this complex intervention in neonatal intensive care units (NICUs) across England.

**Design:**

Process evaluation and qualitative study using a standard questionnaire and interviews. Normalisation Process Theory (NPT) core constructs were used to assess relevant issues to staff embedding ‘CoolCuddle’, to discern change over time and different settings. Qualitative interviews provided valuable contextual exploration of implementation.

**Setting and participants:**

Six tertiary NICUs in England. Thirty-seven families with a newborn baby undergoing cooling therapy for HIE were recruited from September 2022 to August 2023; 17 NICU staff Normalisation MeAsure Development (NoMADs) at six NICUs over 6 months were included; 14 neonatal/research nurses from three participating NICUs were interviewed.

**Intervention:**

The family-centred intervention ‘CoolCuddle’ was developed to enable parents to hold their infant during cooling, without affecting the cooling therapy or intensive care.

**Outcome measures:**

NoMAD questionnaires at three timepoints over 6 months and NPT informed qualitative interviews.

**Results:**

NoMAD questionnaires at baseline showed more variation between units, for intervention acceptability, than those at 3 and 6 months. Qualitative data highlighted that staff understood the benefits of CoolCuddle but were apprehensive due to perceived risks involved in moving cooling babies. A rigorous standard operating procedure was flexible enough to incorporate the use of local processes and equipment and provided the relevant procedural knowledge to deliver CoolCuddle safely.

**Conclusions:**

The CoolCuddle intervention can be implemented safely under the supervision of standard neonatal teams as part of usual practice in diverse NICU settings in England. The importance of having a rigorous standard operating procedure, which can be adapted to support local settings, is highlighted.

**Trial registration number:**

ISRCTN10018542; Results: registered on 30 August 2022.

**STRENGTHS AND LIMITATIONS OF THIS STUDY**

- This study used normalisation process theory to both guide and measure the process of implementation of CoolCuddle in diverse neonatal units across England with different patient populations.
- Normalisation MeAsure Development questionnaires were used to follow the implementation process over 6 months combined with staff interviews to add depth to questionnaire findings.
- This mixed methods approach helped to understand barriers, facilitators and contextual factors impacting the embedding of CoolCuddle in tertiary neonatal intensive care units in England.
- The cohort methodology brings with it the limitations of observational research, although it allowed the implementation process to be studied more closely and in ‘real-world’ conditions.

## Introduction

 Newborn infants exposed to lack of oxygen and blood flow to the brain around birth may develop brain dysfunction called hypoxic-ischaemic encephalopathy (HIE). Globally HIE incidence varies in high and low-income countries. In high-income countries, HIE ranges from 1 to 3 cases/1000 live births,^1^,^3^ but in low-income countries, where timely access to neonatal care is more limited, can be as high as 30.6/1000 live births.^4^,^7^ In England and Wales, HIE incidence was recently recorded as 2 per 1000 live term births (≥37 gestational weeks).^2^ HIE is a leading cause of neonatal mortality^2^ responsible for 1 million neonatal deaths per year,^8^,^10^ is the largest contributor to brain injuries among term infants^11 12^ and can result in significant, and persistent, motor, sensory, cognitive and behavioural impairments.^13^,^16^

To reduce mortality and mitigate brain injury, current evidence-based practice for newborn infants with moderate to severe HIE in high-income countries is to receive therapeutic hypothermia and intensive care (hereafter ‘cooling therapy’) in a neonatal intensive care unit (NICU).^817^,^22^ Cooling therapy is started as soon as possible after HIE diagnosis to achieve therapeutic hypothermia within 6 hours of age and continues for 72 hours.^21^ Infants with HIE born at a local neonatal unit or special care baby unit are transferred to an NICU to receive this therapy.^23^

Each year in the UK over 800 babies with neonatal HIE undergo cooling therapy, and, in the UK, usual practice is for parents not to hold their infant during cooling; due to concerns of destabilising the cooling process or intensive care. However, the broader family impact of HIE is significant,^24^,^26^ parent–infant bonding and breast feeding can also be negatively impacted.^27 28^ As impaired parent–infant bonding is associated with cognitive and emotional impairment in childhood, the promotion of early parent–infant interaction for infants undergoing cooling therapy may enhance bonding and potentially improve cognitive development. Therefore, we developed the ‘CoolCuddle’ intervention to enable parents to hold their infant during cooling, without significantly affecting the cooling therapy or intensive care.^29^ CoolCuddle has already been delivered safely, without impacting cooling therapy, as part of usual practice in two tertiary NICUs, under supervision of an advanced neonatal nurse practitioner. Mothers who participated in CoolCuddle(s) reported reduced postnatal depression, and stable mother–infant bonding scores until 8 weeks postpartum.^27 29^ To maximise the health benefits for this patient population, the CoolCuddle intervention needs to be delivered safely in diverse NICU settings, under the supervision of standard neonatal teams, as part of usual practice.

Few evidence-based medical interventions report how a technological process is embedded in a healthcare system, or integrated by staff to become a ‘routine’ part of medical care.^30^ Normalisation process theory (NPT) is a framework that can be used to support the implementation and evaluation of complex interventions.^31^,^33^ NPT was chosen for this process evaluation as it can be used to focus on how staff routinely incorporate a complex intervention in practice and embed it in a specific setting.^34^ NPT is well suited to late-stage translation research, where the primary aim is to accelerate the sustained uptake and integration of an intervention already proven to be effective, such as cooling therapy.^817^,^22^ The aim of this process evaluation and qualitative study was to examine and report the normalisation of a complex family-centred intervention ‘CoolCuddle’, identifying factors that may shape embedding of the intervention in tertiary NICUs in England.

## Methods

### Approach

This process evaluation of embedding CoolCuddle in routine NICU clinical care was informed by the core constructs of NPT (online supplemental table A). These explain what people do rather than their attitudes or beliefs^35^ and allow for comparisons to be made between contexts, mechanisms and outcomes of implementation processes.^36^ The Normalisation MeAsure Development (NoMAD) questionnaire is a 20-item self-report survey instrument underpinned by NPT. NoMAD was used to assess issues of specific relevance to neonatal staff embedding the intervention, such as ‘differentiation’ from past practices and ‘initiation’ of an intervention in a particular context.^37 38^ Responses to each NoMAD item are measured on a Likert scale of 1–5 (1=strongly disagree to 5=strongly agree). NoMAD has good face validity, construct validity and internal consistency^38^ and can be adapted to the intervention under investigation. NoMAD was used in the current study to discern change over time and between different settings.^39^

### Intervention

**Figure 1:**
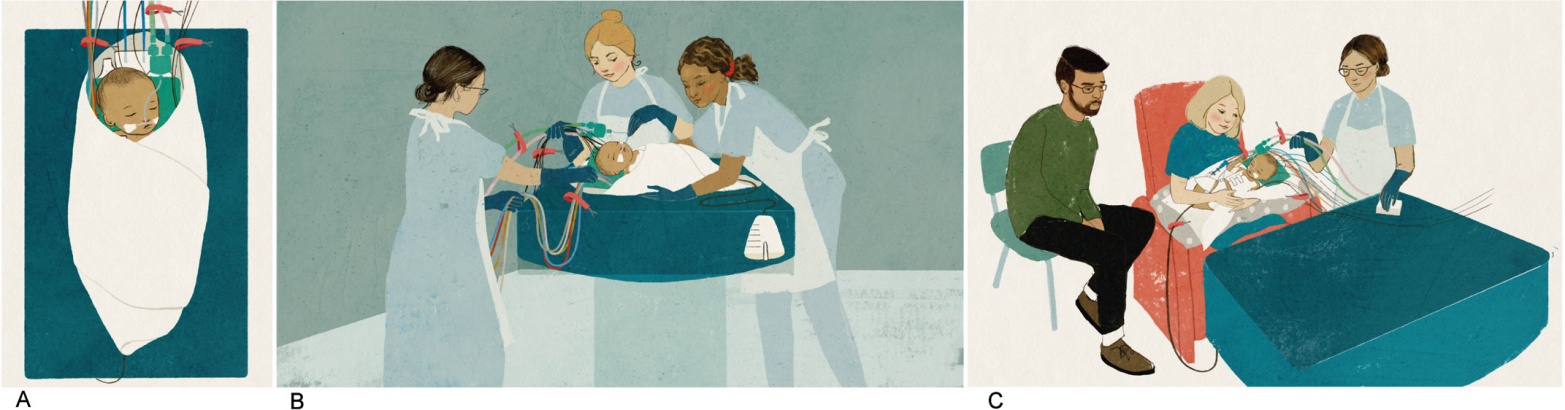
The CoolCuddle intervention. (A) Baby (with attached wires and tubes for the cooling jacket) is wrapped in a sheet. (B) Two or three nurses carefully move baby. (C) To a pillow on the parent’s lap.

The CoolCuddle intervention enabled parents to cuddle and interact with their baby on a pillow on their laps during cooling therapy while nurses closely monitored their baby’s well-being (figure 1 and https://youtu.be/dC7SriN99SA). Before being moved, the wires and tubes around the baby are gathered into two bundles and secured. The baby (including the necessary wires/tubes) is then wrapped in a sheet to keep everything secure (figure 1A) and two or three nurses carefully move the baby (figure 1B) onto the pillow on their parent’s lap (figure 1C). Cuddles can last for up to 2 hours, after which the baby is moved back to their cot.

### Implementation

Six tertiary NICUs participated in the CoolCuddle2 study serving areas with a wide range of patient demographics: Birmingham Women’s and Children’s NHS Foundation Trust, Manchester University Hospitals NHS Foundation Trust, Nottingham University Hospital NHS Trust, South Tess Hospitals NHS Foundation Trust, University Hospitals Leicester NHS Trust and University Hospital Southampton NHS Foundation Trust. All site names have been removed and anonymised (letters A–F) in reporting the process evaluation.

A minimum recruitment of four families per site was considered necessary to study CoolCuddle implementation robustly at each site. From 2022, the number of infants requiring cooling for HIE decreased and so we planned a pragmatic recruitment target of 40 families, since our unit of measurement was the neonatal unit rather than many families. Each neonatal unit had a local principal investigator (PI) and two ‘nurse champions’ (NCs) who led the onsite implementation of CoolCuddle. Online study set up meetings were conducted by the research team (EC, JI, SS); and a training video developed from CoolCuddle1 with a detailed written standard operating procedure (SOP) were included. The SOP was adapted for use by NCs at each NICU to use with existing local procedures and equipment. NCs and local research nurses entered data and logged parental consent using REDCap.^40^ Parents were provided with a short information sheet about the study, and verbal assent was obtained prior to their participation in a CoolCuddle. Before the end of the cooling period, written parental consent was collected to use their data and complete questionnaires.

### Data collection

NICU staff and parents of babies undergoing cooling therapy provided written consent to take part in the study. Babies were excluded from the study if they needed significant cardiorespiratory support (high‐frequency oscillation, mean airway pressure >15 cm H_2_O, oxygen requirement >70%, in situ chest drain, ≥3 inotropes) or status epilepticus at the time of the planned cuddle.

NICU staff completed NoMAD questionnaires adapted for use with CoolCuddle at three timepoints: baseline, 3 and 6 months. Staff were asked questions about their current clinical role and their general views on the implementation of CoolCuddle (online supplemental table B). All questionnaires were sent by email using a secure REDCap hyperlink.^40^ Automatic reminder emails were sent after 1 week if a questionnaire had not been completed.

Interviews and a focus group were conducted by LB (experienced researcher) with neonatal staff at four neonatal units (units A, B, D and F), to expand on findings from NoMAD questionnaires, and explore any differences in implementation between neonatal units. Topic guides (online supplemental table C) were linked to the NoMAD questionnaires. All discussions were recorded and transcribed verbatim by a professional transcription service, and deidentified prior to reporting.

Other data collected (reported elsewhere) include physiology during cuddles, and parent completed questionnaires of postnatal depression, attachment and bonding.^29^ Mothers completed the Edinburgh Postnatal Depression Scale^41^ and the Mother-Infant Bonding Questionnaire^42^ at discharge and 8 weeks postpartum; fathers completed the Paternal Postnatal Attachment Scale^43^ at 8 weeks postpartum; analysis of which is published elsewhere (submitted paper). Interviews were also conducted with parents to explore potential barriers and facilitators for implementing CoolCuddle from a parental point of view.

### Patient and public involvement

Our patient and public involvement advisory group (eight parents with HIE babies) were actively involved in designing parent materials, ways to encourage families to complete questionnaires and interpretation of findings and dissemination to wider audiences. They also gave us feedback on the parent animation about CoolCuddle resulting in some useful additional text being added.

### Data analysis

NOMAD outputs were analysed descriptively and were then cross-referenced with qualitative data from staff interviews at four neonatal units to gain in depth understanding of implementation. Responses to the 20 NoMAD items were analysed as follows: ‘strongly agree’ and ‘agree’ were interpreted as ‘high agreement’ (5 and 4), ‘disagree’ and ‘strongly disagree’ as ‘low agreement’ (2 and 1), and ‘neither agree nor disagree’ as a ‘neutral’ response to the item (3). Measures of the ‘normalisation’ of practice from the NoMAD questionnaires were summarised as mean scores and plotted at baseline, 3 months and 6 months using petal plots, with higher mean scores indicating ‘higher agreement’ with NPT subconstructs.

Qualitative interview and focus group data were organised and coded using NVivo software^44^ and analysed thematically by a qualitative researcher (LB). NPT constructs/subconstructs were then used as an organising thematic structure. Staff interviews were coded chronologically, and candidate themes/subthemes generated inductively. Subthemes relating to the implementation of CoolCuddle were mapped onto NPT constructs deductively using the ‘NPT coding variables—First pass coding manual’ for qualitative researchers.^36^ Coding and themes were discussed with senior qualitative researchers (JI & JH) to achieve consensus and reported to the study management group (JI, DO, EC, JH, DP and SS) by LB, at regular intervals during data collection and analysis.

## Results

From September 2022 to August 2023, the six tertiary NICUs in England recruited 37 families with a newborn baby undergoing cooling therapy for HIE and 60 cuddles took place.

### NoMAD Questionnaires and qualitative data

**Figure 2:**
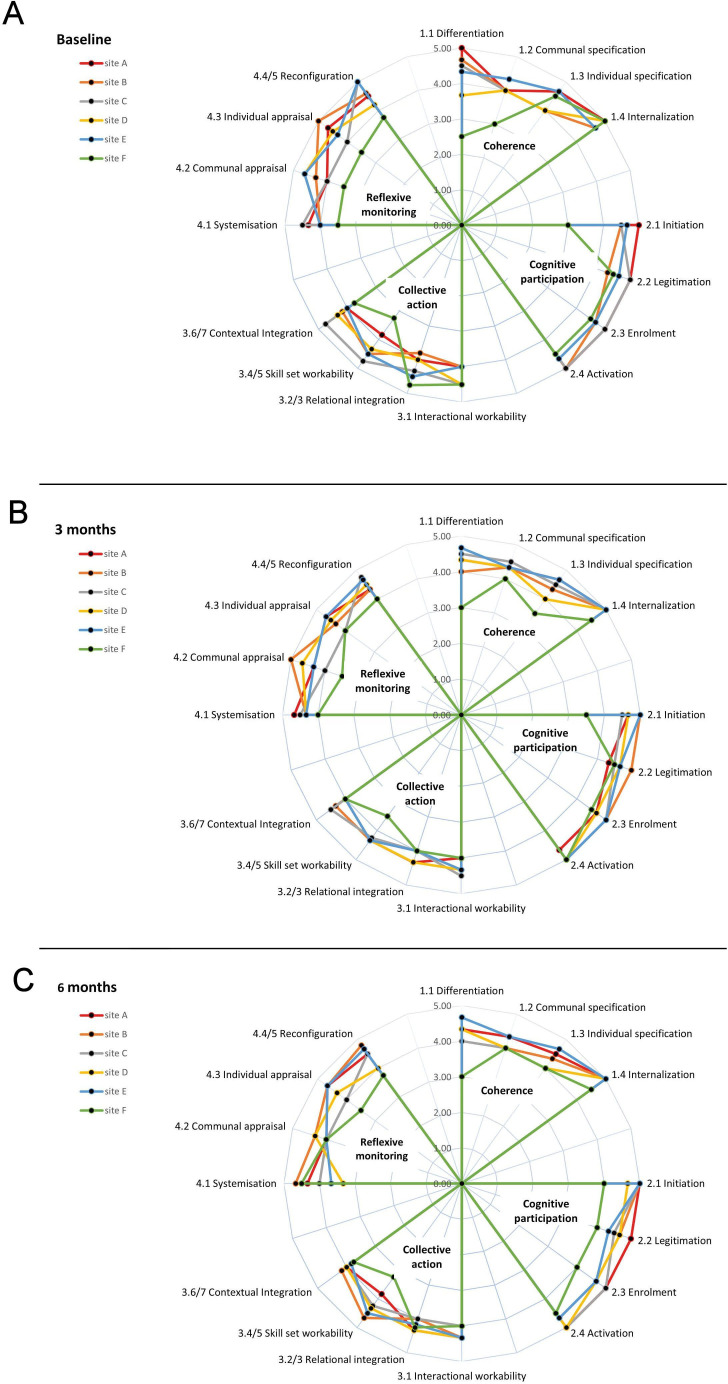
Petal charts showing average scores for the 16 subconstructs of NPT in six neonatal units at three timepoints. (A) Baseline. (B) At 3 months. (C) At 6 months. NPT, normalisation process theory.

NoMAD Questionnaires from 17 NICU staff (15 neonatal nurses and 2 consultant neonatologists) from six neonatal units were included in the data analyses. Demographic data for these respondents are found in online supplemental table D. Mean construct and subconstruct scores are shown in online supplemental tables E–H. NoMAD questionnaires at baseline showed more variation between units, for intervention acceptability, than those at 3 and 6 months. Mean subconstruct scores for each of the six units were plotted at baseline, 3 months and 6 months using petal plots (see figure 2A–C). Petal plots convey variability between sites visually and specifically highlight that site F had relatively lower mean scores from other sites in relation to the ‘reflexive monitoring’ construct, and the subconstructs differentiation, initiation, and skill set workability.

From April to August 2023, eight neonatal/research nurses from three participating neonatal units took part in one-to-one or joint interviews. Six neonatal/research nurses from one further unit took part in a focus group. All neonatal/research nurses who participated in an interview/focus group were active frontline staff in the neonatal units, seven were ‘NCs’ and seven were members of the wider neonatal teams; all delivered the CoolCuddle intervention. Demographic data for these participants are in online supplemental file, table 1. Illustrative quotes from the interviews are shown in tables1^4^, identified by participant number and site.

**Table 1:** Illustrative quotes ‘coherence’

Subconstruct/theme name	Illustrative quotes
1.1 Differentiation*The first cuddle staff think of is a kangaroo cuddle*	*I think people’s aversions to it [CoolCuddle*)*, and the fear of it is because you’re thinking you’re going to do a kangaroo cuddle, which we’re not. It’s a completely different style of cuddle that we need to re-train the unit on, so that we become as confident with a pillow cuddle as we are with a kangaroo cuddle…**#9, site B**Getting(non-cooling)babies out for a cuddle that have got lots of needs in terms of their support and ventilation, is something that we don’t routinely offer families. So for our babies that are being cooled it did feel like that was a good step, it was a good thing for us to be considering to offer. **#15, site A***
1.2 Communal specification*That second or third pair of hands*	*A lot of the newer (nurses) are keen to hear about it and be involved in the process, so they are quite willing to be that second or third pair of hands when you’re getting the babies out… everyone has been quite receptive to it. **#8, site D**There was some hesitancy with some nurses initially on getting the babies out, but actually once we worked through it, got the baby out, and put the baby back, they had nothing but positives to say about it, because it worked really well. #**13, site B***
1.3 Individual specification*Cuddles are nurse led*	*Most of the time it’s very nurse led who drives the thought of a cuddle, and drives the action I would say… **#6, site B**Family integrated care, I think (CoolCuddle) is a good part of that …. it’s a good way of promoting it with babies that are being cooled…Getting the families involved in the baby’s care as much as possible. **#17, site A***
1.4 Internalisation*It’s a lovely thing to be able to offer our families*	*It’s a lovely thing to be able to offer our families, it’s a positive thing, it’s a good thing. **#15, site A**I thought that [CoolCuddle] sounded like a really great thing to do for parents, because normally they don’t get to hold their babies for at least 72 hours. **#8, site D**I thought it sounded really good, because it’s the first 72 hours of life, really important for bonding. **#17, site A***

**Table 2:** Illustrative quotes ‘cognitive participation’

Subconstruct/theme name	Illustrative quotes
2.1 Initiation*It helps when someone’s got an interest in a particular study*	*There’s a couple of [staff] who’ve got a real interest in it as well… it really helps as well when you’ve got [consultants] or [nurses] who’ve got an interest in a particularly study because then they will come and approach [research team*). *That really helps to embed things as well. **#16, site A**I was quite excited… once I heard about this study… when people from (research team) were talking to [research lead] about people they wanted to put forward to help they immediately came to me and I said “Okay yeah, I can do that.” **#8, site D***
2.2 Legitimation*As long as it’s safe*	*One of my colleagues who was a little bit against it, said, “I don’t see the benefit, I feel that the risks far outweigh the benefit, the fact that this baby is ventilated so the tube could come out. This baby had got central lines that could come out… all these risks of getting this baby out for a cuddle when normally we would wait to day five, what’s the difference?” And actually when she then saw a cuddle happening it changed her mind. **#6, site B** it’s not been risky, we’ve not had any adverse events. **#15, site A**I think it’s a massively positive thing… getting babies out for cuddles, as long as it’s safe to do so, it’s something that we should be prioritising on a daily basis. **#14, site D***
2.3 Enrolment*Senior nurse or medical teams buy-in*	*A couple of the cuddles… it was really busy on the unit, and I did ask the consultant if they were happy for me to get the baby out at that time, …they even came up during the cuddle and asked parents, “How are you doing? We really want you to enjoy your cuddle.” So I had quite positive feedback from the consultants… they were actually really supportive. **#13, site B**There have been plenty of occasions where the clinic team have come to us and said, “We’ve got a baby that’s being cooled*.***#15, site A***
2.4 Activation*It’s about changing people’s mindset*	*I do think it’s about changing people’s mindset… I got the criteria out and I went and showed [the consultant] and I said, “There’s nothing on the criteria that prevents this baby coming out for a cuddle.” **#6, site B***

**Table 3:** Illustrative quotes ‘collective action’

Subconstruct/theme name	Illustrative quotes
3.1 Interactional workability*Taking the time to prepare beforehand*	*I think we just did everything quite slowly… wrapped the cooling mattress around baby, put the lines in securely, so we knew that really the chances of anything falling out were low. **#17, site A**It’s taking the time to prepare everything… that’s what the video is really clear in, really prepare everything first, calmly, neatly, and then the actual moving the patient themselves is so easy. **#14, site D***
3.2 Relational integration*Gaining confidence*	*Sometimes there’s some junior staff that may be a little bit more anxious around getting those [cooled] babies out. But if they are being supported by senior members of the team that’s something that is easily overcome as well. **#3, site F**I think for us one of our biggest barriers, is making sure [staff] are confident, and that they know what they’re doing**. #8, site D***
3.3 Skill set workability*Keeping it niche to begin with*	*Only a small amount [are trained] at the moment, probably just a handful… Which is why if we took it forward as a simulation then maybe we could involve more members of staff. **#17, site A**I think there will be a rolling out of the teaching. We were just trying to keep it niche to begin with… it’s definitely fed back that people are mostly really supportive of it, they just would like the teaching, and then it would be more engrained. **#13, site B**Email and video links were sent round to all members of staff… we’ve got iPads, we were going round with those and showing people the mechanics of it… it’s one of these things that ends up cascading training doesn’t it? **#14, site D***
3.4 Contextual Integration*Staff numbers and skill mix*	*I know a lot of the time staffing is an issue with getting babies out for CoolCuddle, so I know there is staffing issues at times when we’ve got a high volume of babies and not enough nurses. **#17, site A**I think every neonatal unit is struggling with the lack of experienced qualified… staff, and that can make things difficult as well, because again patient safety has to be the priority*.***#14, site D***

**Table 4:** Illustrative quotes ‘reflexive monitoring’

Subconstruct/theme name	Illustrative quotes
4.1 Systemisation*Our best interest is always the patient*	*I think potentially what one of the barriers might be the risk vs benefit information. I think because it’s been drilled in from very early point that our central lines are so vital that maybe we need to spell it out what is the risk, and the benefit, so that people are really clear, and can feel confident that we’ve made a really good clinical judgement that this is going to actually help the situation. **#11, site B**I definitely feel that the babies are being monitored, we’re doing everything in a calm way, we’ll always pause or stop if we need to, and that clear expectation that if anything changes we can and will move the baby back. … just that level of reassurance, our best interest is always the patient, and we’re not going to do anything that we feel is putting them at risk**. #14, site D***
4.2 Communal appraisal*We’ve changed to a hot cot to help facilitate this*	*We have a cooling guideline, and we’ve also got a nursing SOP of point by point of what to do, and we’ve added in there about using hot cots. So we’ve got it built into our normal practice moving forward. **#6, site B**I don’t think it’s fully incorporated into the day to day routine yet. That all takes a bit more time for people to really think about it as being the standard care… I thought we’d get more resistance from people, but actually most people have been really keen to be involved and quite happy to get the babies out. **#8, site D***
4.3 Individual appraisal*Seeing the parents’ reactions*	*It’s been one of the most positive things that we could have done for them in the first few days. **#13, site B**When I saw that mum get that baby out… she just looked so happy and so relaxed, and we know that’s going to impact on milk supply, and help with those hormones that she needs to get that milk supply, and honestly she was…. and it was so nice to see. **#16, site A**Mums… talk a lot about it feeling like they haven’t had a baby, because the emergency surgery… they haven’t heard that first cry, so all those things to make their baby feel real to them. Then they get to actually hold their baby… [during CoolCuddle] **#14, site D***
4.4 Reconfiguration*Translating that knowledge*	*When [parents] enter the unit we click a button to say they’re present on the unit, and what pops-up on the screen is an automatic kangaroo cuddle, have you thought of a kangaroo cuddle? to remind the nurse to think about kangaroo. So it might be that we might need to adapt that… to incorporate something like that to move forward. **#6 Site B**I think it’s raised conversations about if we’re offering babies that are being cooled the opportunity to have a cuddle, and they have an arterial line in, why are we not offering other babies with an arterial line the opportunity to come out? So I think it’s opened up conversations why are we not offering that to others? **#15, site A***

### Coherence ‘understanding and opinion of the intervention’

‘Coherence’ construct mean scores ranged from 3.75 to 4.67 at all three time points, suggesting an overall positive opinion of the intervention (online supplemental table E). All respondents ‘strongly agreed’ or ‘agreed’ with the ‘internalisation’ subconstruct, showing a clear understanding of the potential value of the intervention throughout implementation. ‘Communal specification’ and ‘individual specification’ subconstructs ranged from 3.00 to 4.67 across all three timepoints, suggesting that teams developed a shared understanding of the purpose of CoolCuddle early in the implementation process which was sustained as the study progressed. There were noticeably lower scores for ‘differentiation’ at one neonatal unit (site F) across all timepoints (online supplemental table E). Reasons for this are described via focus group and interview data (table 1).

#### The first cuddle staff think of is a kangaroo cuddle—(differentiation)

Site F staff were already allowing cuddles for some, but not all cooling infants. This practice was not ‘protocolised’ and the PI stated: *the CoolCuddle SOP just formalised what we were doing and made it more routine’.* All other participating NICUs had not previously taken babies out of their cot during cooling therapy. Staff in one unit felt it was important to think about a ‘CoolCuddle’ as conceptually different from a ‘kangaroo cuddle’, which involves holding a baby skin-to-skin, placed on a parent’s bare chest. Staff at this unit began to normalise CoolCuddle by defining it as a specific type of ‘pillow cuddle’ and stressed that future training should integrate this important difference, to allay any initial anxieties or safety concerns about CoolCuddle if perceived to be similar to kangaroo care (see the section 'As long as it’s safe'). In contrast, another unit normalised CoolCuddle by comparing it to the existing practice of facilitating cuddles for other groups of ‘high-risk’ ventilated babies with multiple lines.

#### That second or third pair of hands—(communal specification)

Where CoolCuddle was a new practice, staff demonstrated how they worked as a team to understand it and viewed it positively as aligning with existing unit philosophy of encouraging cuddles as part of wider family-centred care practices. NCs or senior neonatal nurses helped other staff integrate CoolCuddle into usual practice, particularly those who were hesitant about moving babies while receiving cooling therapy. They shared knowledge about CoolCuddle’s purpose and involved less experienced team members in observing and acting as a second or third pair of hands when moving a cooled baby.

#### Cuddles are nurse led (individual specification)

Neonatal nursing staff took individual responsibility for CoolCuddle since it helped with ensuring delivery of family-centred care to facilitate parent-infant bonding as soon as possible after birth. The intervention was compared with existing similar nurse-led good practice on the unit, including skin-to-skin and kangaroo care.

#### It is a lovely thing to be able to offer our families (internalisation)

Despite some initial apprehensions about moving babies, the intervention was internalised as nursing staff saw the potential value of CoolCuddle, to enable parents to hold and bond with their baby during their first days of life.

### Cognitive participation ‘engagement with the intervention’

At baseline, 3 and 6 months, the ‘cognitive participation’ construct received the most positive overall response, suggesting high engagement with CoolCuddle at all six units (online supplemental table F). The lowest cognitive participation scores were in site F, particularly in relation to ‘initiation’, since staff were already moving some babies during cooling therapy (see the section 'The first cuddle staff think of is a kangaroo cuddle'), therefore key staff were unlikely to be working to drive CoolCuddle forward.

#### It helps when someone’s got an interest in a particular study (initiation)

Three neonatal units (sites A, B and D) reported that NCs were actively involved in driving the intervention forward, and their enthusiasm was key in engaging other staff members.

#### As long as it is safe (legitimation)

It was important for nursing staff to feel confident that CoolCuddle could be implemented safely to be accepted as a legitimate part of ongoing routine practice. One interviewee reported that a colleague who initially did not see CoolCuddle as a legitimate part of their role, due to the perceived risks of moving a cooling infant outweighing benefit, changed their mind when observing that the intervention could be delivered safely. Interviewees from all units discussed safety as a key part of their practice when delivering care (see also the sections 'Senior nurse or medical teams buy-in', 'Enrolment'; 'Interactional workability'; 'Relational integration' and 'Contextual integration').

#### Senior nurse or medical teams buy-in (enrolment)

Nursing staff worked to build communal engagement with CoolCuddle by sharing clinical information about babies who might be eligible for the study as soon as possible on admission. They discussed ways to involve colleagues to deliver CoolCuddle safely, including gaining support from more senior colleagues when NICU was busy prior to proceeding or ensure they had enough staff to carry out CoolCuddle.

#### It is about changing people’s mindset (activation)

NCs sustained the momentum of embedding CoolCuddle by addressing senior staff concerns who were less familiar with the eligibility criteria and clinical care necessary during a CoolCuddle. One unit included CoolCuddle information in a monthly newsletter to introduce the study to staff more widely.

### Collective action ‘putting the intervention into practice’

‘Collective action’ subconstruct mean scores were more ‘neutral’ (neither agree nor disagree) at all three time points across all units than any of the ‘coherence’ or ‘cognitive participation’ subconstruct mean scores (online supplemental table G). There were also more missing data apparent in raw participant data (online supplemental table J).

Interview data highlight factors that may have contributed to these responses, including the potential impact of the number of Coolcuddle trained staff, staff shortages and high staff turnover (table 3).

#### Taking the time to prepare beforehand (interactional workability)

Staff described interactions prior to each CoolCuddle, methodically planning and preparing each stage of the process, and sharing past experiences with other staff, such as keeping the cot close to the cuddle chair in case the baby needed to be returned to their cot quickly. One potential barrier to CoolCuddle was the impact on their already demanding workload, as moving babies from cot to parent took time and involved several members of trained staff to do it safely.

#### Gaining confidence (relational integration)

CoolCuddle was operationalised by developing team confidence in delivering it via repeated use. Interviewees from all units discussed the importance of involving junior staff, so that they could gain confidence and practical experience, particularly since ‘non-routine’ tasks were involved such as disconnecting the cooling jacket and carrying out necessary safety checks. Staff felt that it was also important for them to convey confidence and competence to parents, so they in turn felt confident to hold their baby during cooling therapy.

#### Keeping it niche to begin with (skill set workability)

The pace of practical training (eg, observing or supporting a CoolCuddle) varied at different units; two decided to train a small number of individuals to deliver CoolCuddle. Unit D reported the highest number of staff trained (20–30), and they had also included specific CoolCuddle training in annual mandatory training. In contrast sites A and B restricted training initially to a limited number of staff; at site A this included NCs and junior staff, at site B NCs trained a small group of senior nursing staff who conducted all CoolCuddles. Interviewees from this unit felt that integrating CoolCuddle in this way caused minimal disruption to working relationships since they were ‘trusted’ members of neonatal staff, and others accepted the intervention because they were delivering it.

#### Staff numbers and skill mix (contextual integration)

Nursing staff reported that the SOP developed by the CoolCuddle clinical team (EC, DO) provided the relevant procedural knowledge to deliver CoolCuddle safely. Two units modified the existing SOP slightly and their current practices to accommodate new equipment needed to deliver CoolCuddle. All neonatal units discussed current staff shortages and high staff turnover as contextual factors, which impacted on the CoolCuddle delivery. To deliver the intervention safely, up to three members of nursing staff with relevant skillsets are needed initially when the baby is moved from the cot to their parents’ arms. Other difficulties included having the time to train junior staff, and the impact of shift patterns, particularly where some staff worked only night shifts.

### Reflexive monitoring ‘appraisal of the intervention’

Nine ‘reflexive monitoring’ subconstruct mean scores were also lower than 4, at all time points (online supplemental table H); again, with more neutral responses and missing data apparent in raw participant data (online supplemental table J).

However, both staff and parent accounts, of either delivering or participating in CoolCuddle, were very positive, and these accounts were reflected on to expand and take the intervention forward in future (table 4).

#### Our best interest is always the patient (systemisation)

Staff appraised CoolCuddle by monitoring formal clinical evaluations of a baby’s condition (at eligibility, during and after the intervention) and by reflecting on informal positive appraisals from parents. Existing medical issues, babies being well enough to take part in or continue with a CoolCuddle and monitoring their physiological stability during the cuddle were all important clinical evidence of whether the intervention was ‘fit for purpose’. However, some staff wanted more information about the risk of dislodging central lines and evidence-based findings on whether the intervention is beneficial for parents and infants.

#### We have changed to a hot cot to help facilitate this (communal appraisal)

Interviewees also reflected on the need to understand why certain aspects of care needed to change to take CoolCuddle forward, such as the use of different equipment (longer lines, a different type of cot). Although CoolCuddle had not yet been fully accepted as standard practice for cooling babies, most staff were keen to be involved and felt it was worth implementing, with units already making changes to the existing SOP and seeking local governance approval for changes to equipment and procedures.

#### Seeing the parents’ reactions (individual appraisal)

Staff reflected on their own experience of delivering or watching families take part in CoolCuddle, highlighting the personal and professional satisfaction they got from observing participating families. They commented on the way CoolCuddle helped parents establish a sense of normality and contributed positively to establishing breast feeding. Staff also appraised the intervention in terms of the value parents placed on it, as they observed parents’ positive reactions.

#### Translating that knowledge (reconfiguration)

Staff also highlighted aspects of CoolCuddle procedure that might be reconfigured in future, such as having pops-up to remind nurses about the possibility of CoolCuddle eligibility. To enhance and provide equity of neonatal care, staff considered modifying practices, so that other high-risk infants could benefit from closer contact with their parents.

## Discussion

Our previous study showed that parents cuddling their babies during cooling therapy enhanced parent–infant bonding and family‐centred care in NICU and was positively received.^27^ This process evaluation reports how the CoolCuddle intervention was embedded in diverse NICUs, caring for families from varied demographic backgrounds in England, using NPT and NoMAD questionnaires to map the implementation process. The NoMAD questionnaires reported more variation between units, in relation to intervention acceptability, at the start than at 3 and 6 months. NPT informed qualitative interviews, and focus groups provided valuable contextual exploration of processes involved in embedding this complex new intervention and expanded on NoMAD questionnaire findings.

After 6 months, the units where CoolCuddle was a new intervention were more positive in relation to most NPT subconstructs, apart from questions on how best to scale-up training further (skill set workability), staffing issues (contextual integration) and determining how effective the intervention is for parents and infants (systematisation). These findings suggest that there are still implementation issues to address, particularly in relation to when and how to scale-up training for larger numbers of staff who work on varied shifts, and how to inform staff of evidence-based findings when an intervention is beneficial.

While NICU staff understood the potential benefits of enabling parents to hold their baby during cooling (internalisation), aligning with existing NICU ‘family centred’ care practices, some were apprehensive about putting the intervention into practice; due to perceived risks involved in moving cooling babies. However, a rigorous but locally flexible SOP, appeared to provide senior NICU staff with the procedural knowledge to deliver CoolCuddle safely. Enthusiastic NICU NCs initiated, and sustained, the momentum of embedding CoolCuddle and wanted to continue using CoolCuddle in future. They addressed staff concerns and answered questions about necessary changes to nurse-led clinical care; supporting team confidence in the novel tasks associated with CoolCuddle (eg, disconnecting the cooling jacket). Junior staff were offered practical support from senior colleagues, and teams discussed how current practice could be modified (eg, using different equipment). CoolCuddle was further embedded through practical experience as neonatal staff at all levels began to accept the intervention as safe, and observed parents’ positive reactions to it, making them more likely to use the intervention in the future (see legitimation, enrolment and activation). Staff had begun to appraise the intervention and considered the potential for modifying their practices further in future, for example, to facilitate parent–infant cuddles for other high-risk babies.

Studies of implementation of new techniques or processes in NICUs, including those enhancing family-integrated care, have often focused on using qualitative interviews alone or with a parent behaviour change questionnaire (such as self-efficacy measurements) in their process evaluations.^27 45 46^ However, studies measuring changes in behaviour of staff while implementing new techniques are less common. Johnson *et al*^47^ used an NPT framework to guide implementation and embedding of new nutrition guidelines for preterm infants in neonatal intensive care. NPT questionnaire scores were compared with guideline compliance audits and concluded that NPT was an effective way of implementing new practices, leading to sustained changes in care in NICU. Redwood *et al*^48^ conducted a qualitative process evaluation of a quality improvement (QI) strategy comparing a standard or enhanced QI support package, to scale up a clinical intervention to increase the administration of magnesium sulphate to women in preterm labour. Similar to the CoolCuddle process evaluation, this study also collected data via interviews with key individuals in leadership positions in maternity units in England and drew on NPT constructs at the analysis stage, to explore how different contexts and team dynamics contributed to implementation outcomes. Redwood *et al* found that ‘normative restructuring’ in units (eg, changes to existing norms, rules and resources) enabled magnesium sulphate to be administered irrespective of whether a unit received the standard or enhanced support package.^48^ However, ‘relational restructuring’ (eg, changes in the way staff related to each other, such as the effective coordination of professionals from different specialties) was more likely to be achieved in units receiving the enhanced support package and was key to facilitating change, so that collective action could be taken and was sustained. The CoolCuddle study also highlighted the importance of having a flexible approach to training materials and supporting local teams to translate changes in their own local context and equipment.^48 49^ Finally, Sutton *et al*^50^ found NPT to be a valuable tool to employ when exploring processes of implementation of a surgical intervention into practice, and in particular using NPT in analysis to recognise the importance of *coherence* work to successful implementation. They also highlighted the importance of considering implementation processes across all four NPT core constructs when exploring barriers and facilitators to embedding interventions into practice. As with the CoolCuddle intervention, both Sutton and Redwood found that using staff ‘champions’ was beneficial in driving implementation of their interventions.^48 50^

In this work, staff reported the different ways in which they trained staff (eg, practical cascade training, inclusion of information in mandatory training modules) using study provided training videos, and a written SOP. However, the number of staff who were trained was relatively low in two neonatal units, one of which suggested that ‘simulation’ training may be a good way to scale-up the training. This highlights that mode of training should be tailored to each implementing unit and can be varied depending on team needs. Other neonatal units are starting to use our online resources for staff training to enable safe use of CoolCuddle. Future implementation research might use NoMAD questionnaires to drill down on different modes of training delivery, distinguishing between which are more acceptable and effective, and the role in extending ‘CoolCuddle’ to other patient groups in NICU.^51 52^ In contrast, where infrastructure and implementation of safe CoolCuddle may be more challenging, this and other studies support wider closer contact between the family and the infant, to aid bonding with the family, with parental benefits, and little impact on the efficacy of the TH process.^53^

### Strengths and limitations

This was a large study conducted in diverse neonatal units across England with different patient populations. Although only 37 families were involved in the study, 60 cuddles took place in six NICUs, providing numerous opportunities for staff to deliver and refine the intervention in their local settings on multiple occasions. Due to the rapid turn-over of new frontline staff in NICUs globally, our findings suggest that CoolCuddle training would need to be repeated regularly. Other neonatal units are starting the use our resources to train staff and keep the SOP in NICU to ensure safe practice.

We used the NoMAD questionnaire to follow the implementation process, and interviews/focus group with staff from four NICUs added depth to the questionnaire findings and helped us understand barriers, facilitators and contextual factors impacting the embedding of CoolCuddle in tertiary NICUs in England. We invited three or four staff members in each unit to complete NoMAD questionnaires every 3 months, after discussing with colleagues at each time point how well the intervention was being integrated into practice. They then completed the questionnaire with their colleagues’ responses in mind. To the best of our knowledge, we have not seen this way of administering NoMAD questionnaires to date in the literature and found it to be a successful way to monitor the implementation process and gain consistent responses about how it differed from previous practice.^34^

However, we were only able to interview staff from four of the six participating NICUs due to variation in the timing that each unit received R and D approvals. The cohort methodology also brings with it the limits of observational research, although it allowed the implementation process to be studied more closely and in ‘real-world’ conditions.

## Conclusions

Providing safe opportunities for parents to bond with their newborn infant remains important, and family-centred care practices such as CoolCuddle give parents the opportunity to participate in the planning, and delivery, of care for their baby at a key time, and after significant trauma. This study establishes that the CoolCuddle intervention can be implemented safely across the England, under the supervision of standard neonatal teams as part of usual practice in diverse NICU settings in England. It highlights the importance of having a rigorous SOP, with flexibility to incorporate local processes and equipment, alongside comprehensive training resources such as the training (https://youtu.be/dC7SriN99SA) and parent animations (https://youtu.be/ZVN83K0xp7g). These can be used as part of a wider training package and can support and sustain the use of this intervention more widely in future.

## supplementary material

10.1136/bmjopen-2024-088228 online supplemental file 1

## Data Availability

Data are available upon reasonable request.
