# Supplementary material for: Embedding the ‘CoolCuddle’ intervention for infants undergoing therapeutic hypothermia for hypoxic-ischaemic encephalopathy in NICU: an evaluation using normalisation process theory
Source: BMJ Open. 2024 Oct 18;14(10):e088228. doi: 10.1136/bmjopen-2024-088228 (PMC11492938; doi:10.1136/bmjopen-2024-088228)

## Table A: NPT Constructs, and sub-constructs

| **Coherence:**  Understanding and opinion of the intervention  *(NoMAD items 1-4)* | **- Differentiation:** Understand how the CoolCuddle intervention is different from (past) practices.  **- Communal specification:** Working together as a team to build a shared understanding of the aims, objectives, and expected benefits of CoolCuddle.  **- Individual specification:** Individual understanding of specific tasks and responsibilities relating to CoolCuddle.  **- Internalisation:** Understanding the value, benefits and importance ‘worth’ of CoolCuddle. |
| --- | --- |
| **Cognitive participation:**  Engagement with the intervention  *(NoMAD items 5-8)* | **- Initiation**  Key participants are working to drive CoolCuddle forward.  **- Legitimation**  Staff believe it is right for them to be involved, and that they can make a valid contribution.  I believe that participating in CoolCuddle is a legitimate part of my role.  **- Enrolment**  Staff may need to organise or reorganise themselves and others in order to collectively contribute to the work involved in delivering CoolCuddles in NICU.  **- Activation**  Staff need to collectively define the actions and procedures needed to sustain Coolcuddle. |
| **Collective action:**  Putting the intervention into operation  *(NoMAD items 9-15)* | **- Interactional workability:** Interactions between staff as they work to integrate CoolCuddle.  **- Relational integration:** Knowledge that staff acquire to build accountability and maintain confidence in CoolCuddle, and in each other as they use the intervention.  **- Skill set workability:** The division of labour that is built up around CoolCuddle as it is are operationalised in NICU.  **- Contextual Integration:** The allocation of different kinds of resources and the execution of protocols, policies and procedures in relation to CoolCuddle. |
| **Reflexive monitoring:**  Appraisal of the intervention  *(NoMAD items 16-20)* | **- Systemisation:** Determining how effective and useful CoolCuddle is for staff, parents, and infants by collecting formal and informal information associated with the intervention.  **- Communal appraisal:** Working together as a team to evaluate the worth of the intervention.  **- Individual appraisal:** Working experientially as individuals to express their personal relationship to the intervention, and the effect it has on their work.  **- Reconfiguration:** Attempts to redefine procedures, modify practices, or change the shape of the intervention. |

## Table B: CoolCuddle2 adapted NoMAD questionnaire

| **1.** **This survey asks questions about the implementation of CoolCuddle.**  **From the statements below please choose an option that best describes your main role in relation to CoolCuddle:**  I am involved in managing or overseeing CoolCuddle  I am involved in delivering CoolCuddle  **Part A: About yourself**  **2. How many years have you worked for this [name of organisation]?**  Less than one year; 1-2 years; 3-5 years; 6-10 years; 11-15 years; More than 15 years  **3. How would you describe your professional job category?**  Neonatal nurse  Neonatal consultant |
| --- |
| **Part B: General questions about CoolCuddle**  **1. Do you feel CoolCuddle is currently a normal part of your work?**  Not at all Somewhat Completely  0 1 2 3 4 5 6 7 8 9 10  **2. Do you feel CoolCuddle will become a normal part of your work?**  Not at all Somewhat Completely  0 1 2 3 4 5 6 7 8 9 10 |
| **Part C: Detailed questions about CoolCuddle**  Strongly agree Agree Neither agree nor disagree Disagree Strongly disagree  5 4 3 2 1  *Coherence**  1. I can see how CoolCuddle differs from usual ways of working  2. Staff in this organisation have a shared understanding of the purpose of CoolCuddle  3. I understand how CoolCuddle affects the nature of my own work  4. I can see the potential value of CoolCuddle for my work  *Cognitive participation**  5. There are key people who drive CoolCuddle forward and get others involved  6. I believe that participating in CoolCuddle is a legitimate part of my role  7. I’m open to working with colleagues in new ways to use CoolCuddle  8. I will continue to support CoolCuddle  *Collective action**  9. I can easily integrate CoolCuddle into my existing work  10. CoolCuddle disrupts working relationships  11. I have confidence in other people’s ability to use CoolCuddle  12. Work is assigned to those with skills appropriate to CoolCuddle  13. Sufficient training is provided to enable staff to use CoolCuddle  14. Sufficient resources are available to support CoolCuddle  15. Management adequately support CoolCuddle  *Reflective monitoring**  16. I am aware of reports about the effects of CoolCuddle  17. The staff agree that CoolCuddle is worthwhile  18. I value the effects CoolCuddle has had on my work  19. Feedback about CoolCuddle can be used to improve it in the future  20. I can modify how I work with CoolCuddle |

**NPT construct headings were not included on the questionnaire.*

## Table C: Topic Guide

| **Interview/Focus group objectives:** We would like to understand how clinical teams have integrated CoolCuddle into their day-to-day clinical practice, and explore your views and experiences of the intervention.  **Introduction****s:** Researcher/interviewee(s)  Years experience in current & other NICUs?  Current responsibilities relating to CoolCuddle2?  **Coherence: Understanding and opinion of the intervention**   1. What are your general views on providing CoolCuddles for families?   *[Prompts: *NOMAD was this a completely new practice in your unit, or have parents been holding babies during cooling therapy before the study started?]*  **Cognitive participation: Engagement with CoolCuddle**   1. Has the use of CoolCuddle changed any aspects of clinical practice?   *[Prompts: what/how? any modifications needed e.g. SOP/equipment?]*   1. Has anything helped embed CoolCuddle?   *[Prompts: what/how?]*   1. What kind of barriers have been encountered?   *[Prompts: what/able to resolve?]*  **Collective action: Putting CoolCuddle into operation**   1. How has your team incorporated CoolCuddle into day-to-day practice?   *[Prompts: how many staff have been trained? *NoMAD sufficient training?]*   1. Do you have confidence as a team in your ability to continue to provide CoolCuddle? *[Prompts: scaling up?* **NoMAD disruption to working relationships how/why?]* 2. Any staff feedback on CoolCuddle, which might improve the process in the future? 3. We will be producing a new training video (animation, 8 minutes) for future use, any suggestions for what should be included? 4. Also producing a short 2 minute animation for parents – how easy would it be to use this with parents?   *[Prompts: give parent QR code or weblink to use on their mobile?]*    **Reflective monitoring: Appraisal of CoolCuddle**   1. Do you have any feedback about assessing the effectiveness of CoolCuddle?   *[Prompts: *NoMAD any reports about the effects of CoolCuddle? the effects CoolCuddle has had on your work? *NoMAD can staff modify how they work with CoolCuddle? How - SOP/ moving baby/ on NICU?]*   1. Any feedback from parents about CoolCuddle, which might improve the process in the future?   [*Prompts: What questions have parents had? (before, during, after cooling) any safety concerns?]*   1. Do you have any further comments or thoughts about what we have discussed?   **NoMAD - Prompts added after NoMAD questionnaire feedback @ baseline/3 months.*  **Thank you for your time.** |
| --- |

## Table D: NoMAD Questionnaire respondent demographics

## Table E. ‘Coherence’ construct and sub-construct mean scores at 3 timepoints

## Table F: ‘Cognitive Participation’ construct and sub-construct mean scores at 3 timepoints

## Table G: ‘Collective Action’ construct and sub-construct mean scores at 3 timepoints.

## Table H: ‘Reflexive Monitoring’ construct and sub-construct mean scores at 3 timepoints

## Table I: Interview/focus group participant demographics

## Table J: Raw NoMAD scores by participant
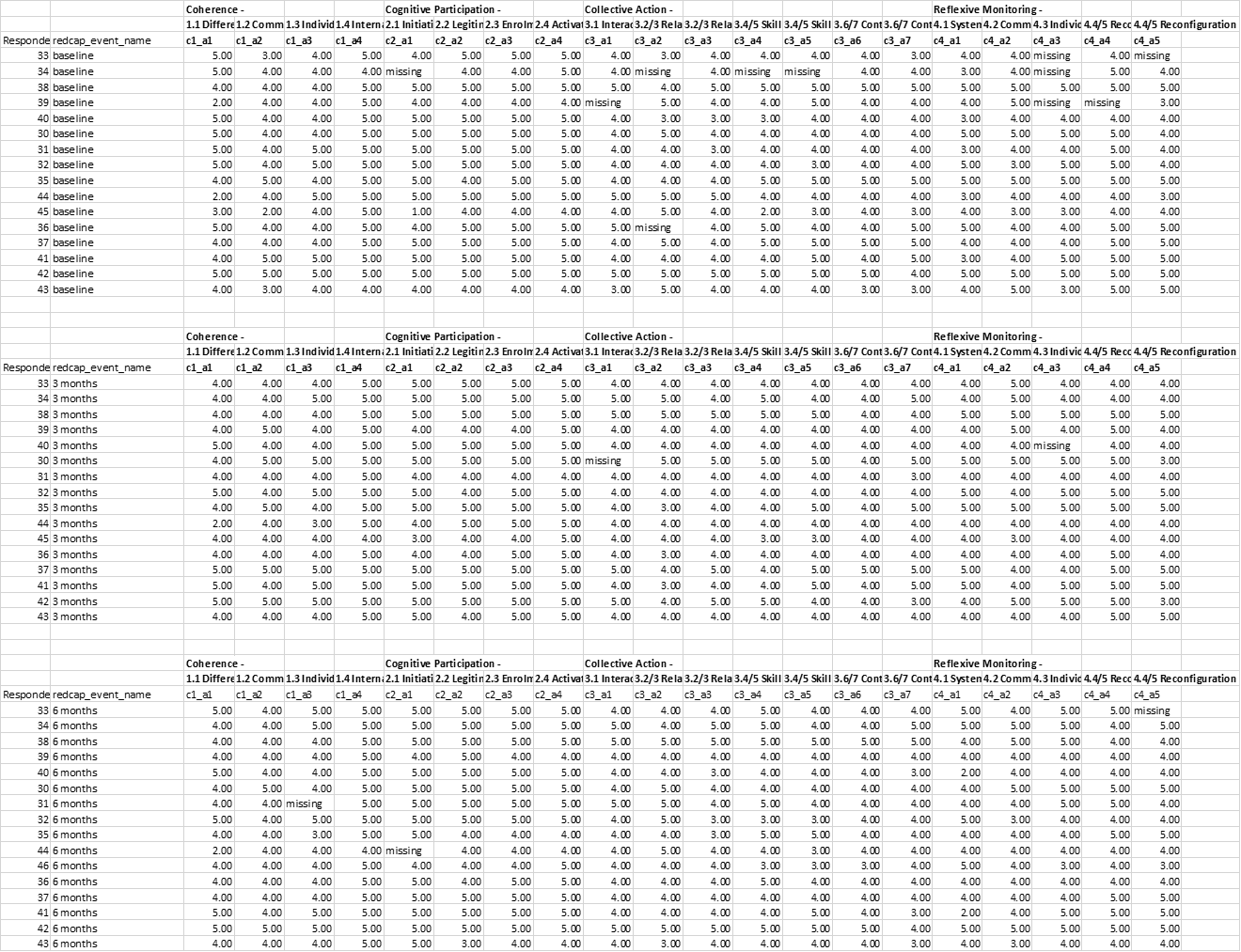

Supplement: online supplemental file 1 [file bmjopen-14-10-s001.docx]
