# Supplementary material for: Embedding the ‘CoolCuddle’ intervention for infants undergoing therapeutic hypothermia for hypoxic-ischaemic encephalopathy in NICU: an evaluation using normalisation process theory
Source: BMJ Open. 2024 Oct 18;14(10):e088228. doi: 10.1136/bmjopen-2024-088228 (PMC11492938; doi:10.1136/bmjopen-2024-088228)
Supplement: Supplementary file 2 [file bmjopen-2024-088228.reviewer_comments.pdf]

PEER REVIEW HISTORY

BMJ Open publishes all reviews undertaken for accepted manuscripts. Reviewers are asked to complete a checklist review form and are provided with free text boxes to elaborate on their assessment. These free text comments are reproduced below.

ARTICLE DETAILS

Title (Provisional)

Embedding the ‘CoolCuddle’ intervention for infants undergoing therapeutic hypothermia for hypoxic-ischaemic encephalopathy in NICU: an evaluation using normalisation process theory

Authors

Beasant, Lucy; Chakkarapani, Ela; Horwood, Jeremy; Odd, David; Stocks, Stephanie; Parker, Denise; Ingram, Jenny

---

VERSION 1 - REVIEW

**Reviewer**	**1**
**Name**	**Afifi, Jehier**
**Affiliation**	**Dalhousie University, Pediatrics**
**Date**	**09-Aug-2024**
**COI**	**No conflict of interest to disclose.**

This mixed method study used Normalization Process Theory (NPT) to guide and evaluate the implementation process of CoolCuddle intervention during therapeutic hypothermia across six tertiary neonatal units in England. Evaluation of the implementation process in this manuscript focused on understanding how normalisation of a new complex parent-centred intervention was “embedded” in different NICUs caring for patients’ populations and families from diverse demographic backgrounds. The authors used NPT informed qualitative interviews and focus groups and the NoMAD questionnaires over 6 months to assess the implementation process and the variability in adaptation and sustainability across different practices and cultures. The valuable information generated by these methods provided contextual evaluation of how CoolCuddle can be embedded into practice as “standard of care” and identified how acceptability, feasibility and sustainability, including barriers and facilitators, varied based on local context.

The manuscript is well written following “Consolidated criteria for reporting qualitative studies (COREQ)”. A convenience sample of 37 families and 17 staff for qualitative interviews over 6 months was selected. The methods explained how NPT constructs were used as

organizing thematic structure to guide interviews and the theoretical induction of major themes and subthemes. The results section focused on themes and quotes for illustration (page 9-16) and the discussion section clarified induced themes and highlighted diverse or variable results. References and supplementary materials were adequate.

The following are suggestions for authors to improve readability and understanding of this study:

1- The authors to explain why interviews targeted research staff and project champions rather than the frontline staff who are impacted by the practice change. Why attempts to recruit staff from the two non-participating sites failed also worth explanation.

2- The results section page 8 to describe the main findings from the mean scores of NPT questionnaires constructs and subconstructs (Tables D, E, F, G), highlighting the difference by site (e.g. site F not only for initiation but also for reflexive monitoring) and by domain (e.g. skill set workability).

3- Page 8 in results to describe findings of the petal plots (see Figure 2 i-iii) in relation to variability over time; the expectation is that the level of agreement increases, and the variability would decrease over time. However, this was minimally different (Page 18 line 393-395).

4- The lack of variability within some domains over time was discussed in the manuscript (page 19 lines 398-410). Engagement strategies and successful practices, apart from SOP and educational videos, that led to frontline staff behavioral change to be highlighted in more depth in the discussion.

5- Being a mixed method study, to balance interpretation and discussion of both the quantitative and qualitative data and to give examples explaining explain "how": the qualitative interviews were used to guide in-depth understanding of NPT questionnaires, to target some of the identified barriers and to individualize changes based on local context.

6- In the limitations, to address how the standardized team of champions and few trained team members may limit the the generalizability of this approach, in view of the rapid turn-over and new frontline staff in NICUs globally.

Reviewer 2

Name Auriti, Cinzia

Affiliation Saint Camillus International University of Health and Medical Sciences, Pediatrics

Date 17-Aug-2024

COI I have no competing interests

Authors:

Title "Embedding the 'Cool Cuddle' intervention for infants undergoing therapeutic hypothermia for hypoxic-ischemic encephalopathy in NICU: a process evaluation using normalization process theory."

Reviewer:

Process evaluation using process theory.... please use a less "convoluted" title……

Abstract, page 3 line 29

Authors: ……birth develop brain dysfunction (hypoxic-ischemic encephalopathy – HIE)

Reviewer: …may develop brain dysfunction…and in this case ……

Line 30

Authors: ….and are not held by their parents.

Reviewer: and in many neonatal care units they are held by their parents….

…but not in all. In several centers, parents stay close to newborns during the therapeutic hypothermia, if newborns are clinically stable. Please revise the sentence.

Line 33

Authors: This process evaluation and qualitative study examined…..

Reviewer: This process of evaluation and qualitative study, considering experiences in other nations in EUROPE,. Examined…..

Kokkonen Nassef S, Blennow Bohlin M, Jirwe M. Experiences of parents whose school-aged children were treated with therapeutic hypothermia as newborns: A focus group study. Nurs Open. 2023 Nov;10(11):7411-7421. doi: 10.1002/nop2.1994. Epub 2023 Sep 26. PMID: 37752681; PMCID: PMC10563413.

Nassef SK, Blennow M, Jirwe M. Parental viewpoints and experiences of therapeutic hypothermia in a neonatal intensive care unit implemented with Family-Centred Care. J Clin

Nurs. 2020 Nov;29(21-22):4194-4202. doi: 10.1111/jocn.15448. Epub 2020 Aug 20. PMID: 32761952.

Quirke F, Ariff S, Battin M, Bernard C, Bloomfield FH, Daly M, Devane D, Haas DM, Healy P, Hurley T, Kibet V, Kirkham JJ, Koskei S, Meher S, Molloy E, Niaz M, Ní Bhraonáin E, Okaronon CO, Tabassum F, Walker K, Biesty L. Core outcomes in neonatal encephalopathy: a qualitative study with parents. BMJ Paediatr Open. 2022 Jul;6(1):e001550. doi: 10.1136/bmjpo-2022-001550. Epub 2022 Jul 25. Erratum in: BMJ Paediatr Open. 2022 Aug;6(1):e001550corr1. doi: 10.1136/bmjpo-2022-001550corr1. PMID: 36053648; PMCID: PMC9328095.

Line 50-53

Conclusions

Authors: The Cool Cuddle intervention can be implemented safely under the supervision of standard neonatal teams as part of usual practice in diverse NICU settings in England. The importance of having ………… , a rigorous standard operating procedure, that can be adapted to support local settings is highlighted.

Reviewer: The importance of implement the staff training by skilled psychologists……, of defining rigorous standard operating procedures…..

page 4 Background line 75

Authors: …..develop brain dysfunction ..

Reviewer: ….May develop….

Page 5 line 86

Authors: ……Cooling therapy is started as soon as possible after HIE diagnosis.

Reviewer: within 6 hours from the hypoxic -ischemic insult … please cite international guidelines…..

Lines 90-91

Authors: currently parents are not routinely permitted to hold their infant during the 72 hours of cooling, due to concerns of destabilizing the cooling process or intensive care…..

Reviewer: In UK

Intervention

Page 6 lines from 131 to 138

Reviewer: The intervention is well written and the video is very well done. I imagine that it can only be aimed at centers that operate in countries with high socio-health development, because it requires a significant presence of dedicated and trained personnel.

Perhaps it could not be necessary to mobilize the child when the presence of the parents next to the cot and the touch could be a less risky and more practicable solution to enhance and sustain the bounding.

Please discuss also some solutions which could be applicable in other health context.

Page 8 lines from 181 to 185

Authors: Mothers completed the Edinburgh Postnatal Depression Scale (EPDS) and the mother-Infant Bonding Questionnaire (MIBS) at discharge and eight-weeks postpartum; fathers ……

Reviewer: It would have been interesting to compare it with a control group by applying the same questionnaire to the mothers and fathers at the same time points. the lack of a comparison of the parents' perception represents to my opinion a limitation of the study.

Discussion page 18

The discussion is repetitive of concepts already exposed. It does not highlight the advantages of the illustrated care

In summary:

This is a very complex study. The reading is difficult to follow, even if the method is rigorous. The discussion is repetitive of concepts already exposed.

It is necessary to simplify the manuscript which appears very long and it could be interesting to include in the discussion papers in which comparisons have been made in the emotional perception of the newborn's illness in parents who were separated or kept close to the child during cooling. It is possible that keeping parents close to the child does not necessarily require his mobilization, especially in cases of respiratory instability. This would make the procedure better exportable.

Please discuss positively or negatively with the explanation.

VERSION 1 - AUTHOR RESPONSE

Please see attached files

Thank you for the very helpful comments from the reviewers which have improved and clarified the paper.	
**Reviewer comment**	**Response**
StaRI checklist for implementation research	This is now attached.
revise the abstract to BMJopen format	The abstract has been revised.
update the final bullet point in the 'Strengths and limitations' section to specify why the cohort methodology was a limitation. Please ensure that all key limitations are highlighted in the 'Strengths and limitations' section, as well as discussed in full in the main text DISCUSSION section.	We have added this point to the strengths and limitations section within the discussion:<br><br>The cohort methodology brings with it the limits of observational research, although it allowed the implementation process to be studied more closely and in 'real world' conditions.
Were topic guides used for the interviews and focus groups? Please include copies of these as supplemental files, citing the supplemental files where the topic guides are mentioned in the main text METHODS section.	The topic guide has now been added to the supplementary file see pgs.3-4: Table C Topic Guide
Reviewer 1.<br>The following are suggestions for authors to improve readability and understanding of this study:<br>1- The authors to explain why interviews targeted research staff and project champions rather than the frontline staff who are impacted by the practice change. Why attempts to recruit staff from the two non-participating sites failed also worth explanation.	Thank you, we have clarified that all the nurses interviewed were delivering CoolCuddle as members of frontline staff. We have added the following sentence on pg.9:<br>All neonatal/research nurses that participated in an interview/focus group were active frontline staff in the neonatal units, seven were 'Nurse Champions' and seven were members of the wider neonatal teams, all delivered the CoolCuddle intervention.<br><br>We have already noted why we only interviewed staff from 4 of the 6 participating sites on pg.22:

	'However, we were only able to interview staff from four of the six participating NICUs due to variation in the timing that each unit received R&D approvals'.
2- The results section page 8 to describe the main findings from the mean scores of NPT questionnaires constructs and subconstructs (Tables D, E, F, G), highlighting the difference by site (e.g. site F not only for initiation but also for reflexive monitoring) and by domain (e.g. skill set workability).	A summary sentence has been added on pg.8:<br>NoMAD questionnaires at baseline showed more variation between units, for intervention acceptability, than those at three and six-months.
3- Page 8 in results to describe findings of the petal plots (see Figure 2 i-iii) in relation to variability over time; the expectation is that the level of agreement increases, and the variability would decrease over time. However, this was minimally different (Page 18 line 393-395).	And on Pg.8:<br>Petal plots convey variability between sites visually, and specifically highlight that site F had relatively lower mean scores from other sites in relation to the 'reflexive monitoring' construct, and the sub-constructs differentiation, initiation, and skill set workability.
4- The lack of variability within some domains over time was discussed in the manuscript (page 19 lines 398-410). Engagement strategies and successful practices, apart from SOP and educational videos, that led to frontline staff behavioral change to be highlighted in more depth in the discussion.	We have added additional detail on Pg.19 in relation to the way staff further engaged with the intervention as they observed that the intervention was safe, and the positive reaction from participating parents:<br><br>CoolCuddle was further embedded through practical experience as neonatal staff at all levels began to accept the intervention as safe, and observed parents' positive reactions to it, making them more likely to use the intervention in the future (see legitimation, enrolment, and activation).
5- Being a mixed method study, to balance interpretation and discussion of both the quantitative and qualitative data and to give examples explaining explain "how": the qualitative interviews were used to guide in-depth understanding of NPT questionnaires, to target some of the identified barriers and	The qualitative interviews were used to gain a more in-depth understanding of NoMAD questionnaire feedback by adding prompts to the topic guide to explore potential barriers highlighted by NoMAD questionnaire feedback at

to individualize changes based on local context.	baseline and 3 months. See supplementary file topic guide:<br>*NoMAD - Prompts added after NoMAD questionnaire feedback @ baseline/3 months.
6- In the limitations, to address how the standardized team of champions and few trained team members may limit the the generalizability of this approach, in view of the rapid turn-over and new frontline staff in NICUs globally.	We have added:<br>Due to the rapid turn-over of new frontline staff in NICUs globally, our findings suggest that CoolCuddle training would need to be repeated regularly. Other neonatal units are starting the use our resources to train staff and keep the in NICU to ensure safe practice.
Reviewer 2.<br>Process evaluation using process theory.... please use a less "convoluted" title......	The title has been amended and the first 'process' removed:<br>Embedding the 'CoolCuddle' intervention for infants undergoing therapeutic hypothermia for hypoxic-ischaemic encephalopathy in NICU: an evaluation using normalisation process theory.
**Abstract,**<br>page 3 line 29<br>Authors: ......birth develop brain dysfunction (hypoxic-ischemic encephalopathy – HIE)<br>Reviewer: ...may develop brain dysfunction...and in this case	This sentence has been amended.
Line 30<br>Authors: ....and are not held by their parents.<br>Reviewer: and in many neonatal care units they are held by their parents....<br>...but not in all. In several centers, parents stay close to newborns during the therapeutic hypothermia, if newborns are clinically stable. Please revise the sentence.	We have expanded the sentence with a more measured comment, and expanded this in the Background.
Line 33<br>Authors: This process evaluation and qualitative study examined.....<br>Reviewer: This process of evaluation and qualitative study, considering experiences in	We agree, that placing the study in context is important, but we are already at the word limit of the abstract. We already refer to the context (UK) in the abstract and have

other nations in EUROPE,. Examined.....<br><br>Kokkonen Nassef S, Blennow Bohlin M, Jirwe M. Experiences of parents whose school-aged children were treated with therapeutic hypothermia as newborns: A focus group study. Nurs Open. 2023 Nov;10(11):7411-7421. doi: 10.1002/nop2.1994. Epub 2023 Sep 26. PMID: 37752681; PMCID: PMC10563413.<br><br>Nassef SK, Blennow M, Jirwe M. Parental viewpoints and experiences of therapeutic hypothermia in a neonatal intensive care unit implemented with Family-Centred Care. J Clin Nurs. 2020 Nov;29(21-22):4194-4202. doi: 10.1111/jocn.15448. Epub 2020 Aug 20. PMID: 32761952.<br><br>Quirke F, Ariff S, Battin M, Bernard C, Bloomfield FH, Daly M, Devane D, Haas DM, Healy P, Hurley T, Kibet V, Kirkham JJ, Koskei S, Meher S, Molloy E, Niaz M, Ní Bhraonáin E, Okaronon CO, Tabassum F, Walker K, Biesty L. Core outcomes in neonatal encephalopathy: a qualitative study with parents. BMJ Paediatr Open. 2022 Jul;6(1):e001550. doi: 10.1136/bmjpo-2022-001550. Epub 2022 Jul 25. Erratum in: BMJ Paediatr Open. 2022 Aug;6(1):e001550corr1. doi: 10.1136/bmjpo-2022-001550corr1. PMID: 36053648; PMCID: PMC9328095.	caveated this work in place there, and hope this is enough to ground the results (which will of course be health-care service specific to some extent). We have extended the discussion with a paragraph about European practice. Thank you. We have added these references to the background.
Line 50-53<br>Conclusions<br>Authors: The Cool Cuddle intervention can be implemented safely under the supervision of standard neonatal teams as part of usual practice in diverse NICU settings in England. The importance of having .... , a rigorous standard operating procedure, that can be adapted to support local settings is highlighted.<br>Reviewer: The importance of implement the	Psychologists were not involved in the CoolCuddle process as it was a nursing-led intervention. We have not amended the conclusions.

staff training by skilled psychologists……, of defining rigorous standard operating procedures…..	
page 4 Background line 75<br>Authors: …..develop brain dysfunction ..<br>Reviewer: ….May develop….	This has been added.
Page 5 line 86<br>Authors: ……~~Cooling therapy is started as soon as possible after HIE diagnosis~~.<br>Reviewer: within 6 hours from the hypoxic - ischemic insult … please cite international guidelines…..	We have amended this sentence to:<br><br>Cooling therapy is started as soon as possible after HIE diagnosis to achieve therapeutic hypothermia within 6 hours of age, and continues for 72 hours[21]
Lines 90-91<br>Authors: currently parents are not routinely permitted to hold their infant during the 72 hours of cooling, due to concerns of destabilizing the cooling process or intensive care…..<br>Reviewer: In UK	We have amended this as suggested.
Intervention<br>Page 6 lines from 131 to 138<br>Reviewer: The intervention is well written and the video is very well done. I imagine that it can only be aimed at centers that operate in countries with high socio-health development, because it requires a significant presence of dedicated and trained personnel.<br>Perhaps it could not be necessary to mobilize the child when the presence of the parents next to the cot and the touch could be a less risky and more practicable solution to enhance and sustain the bounding.<br>Please discuss also some solutions which could be applicable in other health context.	We have added some comments around this to the discussion:<br><br>In contrast, where infrastructure, and implementation of safe CoolCuddle may be more challenging, this, and other outputs from the CoolCuddle programme, support wider closer contact between the family and the infant, to aid bonding with the family, with parental benefits, and little impact on the efficacy of the TH process.[53]
Page 8 lines from 181 to 185<br>Authors: Mothers completed the Edinburgh Postnatal Depression Scale (EPDS) and the mother-Infant Bonding Questionnaire	We agree that the views of parents are important, and we have carried out further work, and more is ongoing, looking at the EPDS etc scores across,

(MIBS) at discharge and eight-weeks postpartum; fathers ......<br>Reviewer: It would have been interesting to compare it with a control group by applying the same questionnaire to the mothers and fathers at the same time points. the lack of a comparison of the parents' perception represents to my opinion a limitation of the study.	and between families. This is outside the remit of this paper and will be published elsewhere as noted at the end of the data analysis section.
Discussion page 18<br>The discussion is repetitive of concepts already exposed. It does not highlight the advantages of the illustrated care	We have added an introductory sentence to the discussion with the conclusion from our previous work:<br><br>Our previous study showed that parents cuddling their babies during cooling therapy enhanced parent-infant bonding and family-centred care in NICU and was positively received.[27]
In summary:<br>This is a very complex study. The reading is difficult to follow, even if the method is rigorous. The discussion is repetitive of concepts already exposed.<br>It is necessary to simplify the manuscript which appears very long and it could be interesting to include in the discussion papers in which comparisons have been made in the emotional perception of the newborn's illness in parents who were separated or kept close to the child during cooling. It is possible that keeping parents close to the child does not necessarily require his mobilization, especially in cases of respiratory instability. This would make the procedure better exportable.<br>Please discuss positively or negatively with the explanation.	We have amended, and shortened the discussion, alongside this, and other comments, and hope the paper is more readable. We have already (as suggested above), referenced the previous work, and added comments around the exportability of CoolCuddle. We hope the paper is now easier to follow.

**** Response to Reviewer - responses_to_reviewers_Sep24_v3.docx ****

**** Response to Reviewer - Response to Editorial office queries 17 Sept.docx ****

VERSION 2 - REVIEW

**Reviewer**	**1**
**Name**	**Afifi, Jehier**
**Affiliation**	**Dalhousie University, Pediatrics**
**Date**	**25-Sep-2024**
**COI**	

Thank you for your responses and changes/edits made making the manuscript clearer and easily followed. I still think that reporting of the results (page 8-17) needs more work. In the methods, please explain what range in the raw scores on table E to H were indicators of "strong" agreement vs moderate vs none (ie: score >4 is strong agreement) agreement. In coreq check list, you indicated page 18 had described the findings including major and minor themes and constructs, however this was a big picture summary of the findings. You may consider a figure summarizing the main 4 constructs and subconstructs findings from the text (1.1 -4.4) and also removing any redundancy or repetition between the result tables and the text.

VERSION 2 - AUTHOR RESPONSE

Thankyou for the further comments which we have discussed as a team. Our responses are attached. We have not made any changes to the submitted text but have uploaded a revised COREQ checklist.

Thankyou for the further comments which we have discussed as a team. Our responses are below. We have not made any changes to the submitted text but have uploaded a revised COREQ checklist.

Rev 1 comment	Response
In the methods, please explain what range in the raw scores on table E to H were indicators of "strong" agreement vs moderate vs none (ie: score >4 is strong agreement) agreement.	There is no specific cut-off value to indicate strong/moderate agreement. Plotting NoMAD scores over time was to illustrate that the means increased towards 5 by 6 months and that some variation between sites had decreased. Tables E-H are mean scores not raw scores which are in the suppl file Table J.

In coreq check list, you indicated page 18 had described the findings including major and minor themes and constructs, however this was a big picture summary of the findings.	The Coreq checklist has been corrected to indicate that this should be pages 9 - 18.
You may consider a figure summarizing the main 4 constructs and subconstructs findings from the text (1.1 -4.4)	We feel that adding a figure summarizing the 4 main constructs and subconstructs would not add much to the paper as the 4 constructs are well established part of NPT.
Removing any redundancy or repetition between the result tables and the text.	The quote tables report the data, supporting the interpretation and analysis, and we feel are needed despite some repetition. The prose itself flows better without all the data placed in the manuscript, and the quote tables need to be understandable as standalone entities.

**** Response to Reviewer - Further reviewers comments and responses.docx ****
